# Going Outside the Gut: Immune Thrombocytopenia Presenting as a Rare Extraintestinal Manifestation of Ulcerative Colitis

**DOI:** 10.7759/cureus.61888

**Published:** 2024-06-07

**Authors:** Ana P Rivera, James R Pellegrini Jr., Tulika Saggar, Melvin V Joy, Atul Sinha, Sandra Gomez, Kaleem Rizvon

**Affiliations:** 1 Internal Medicine, Nassau University Medical Center, East Meadow, USA; 2 Gastroenterology and Hepatology, Nassau University Medical Center, East Meadow, USA

**Keywords:** mesalamine therapy, intravenous immunoglobulin (ivig), thrombocytopenia, extraintestinal manifestations in inflammatory bowel disease, extraintestinal manifestations, autoimmune colitis, hematologic autoimmune disorders, immune thrombocytopenia (itp), (ibd) inflammatory bowel disease, ulcerative colitis (uc)

## Abstract

A 26-year-old male with no significant medical history presented with hematochezia and was diagnosed with ulcerative colitis (UC) accompanied by immune thrombocytopenia (ITP) as an extraintestinal manifestation (EIM) of UC. This case report delves into the uncommon overlap between UC, a subtype of inflammatory bowel disease primarily affecting the colon and rectum, and ITP, an autoimmune condition leading to platelet destruction. The patient's atypical presentation and subsequent positive response to a treatment regimen targeting both UC and ITP underscores the necessity for a thorough and multifaceted diagnostic approach in individuals with UC, especially when faced with non-gastrointestinal symptoms like unexplained thrombocytopenia. The findings from this study enhance the understanding of UC's diverse manifestations and highlight its potential intersection with other autoimmune diseases, advocating for integrated care strategies in managing such intricate clinical cases.

## Introduction

Ulcerative colitis (UC) is a subtype of inflammatory bowel disease (IBD) characterized by persistent inflammation primarily affecting the colon and rectum. This condition results in widespread friability and superficial erosions of the colonic wall and is limited to the mucosa and submucosa [1]. The incidence of UC ranges from 9 to 20 per 100,000 people annually, with a prevalence of 156 to 291 cases per 100,000 people [2]. While gastrointestinal symptoms such as diarrhea, rectal bleeding, abdominal pain, urgency, and tenesmus are common, patients may also experience weight loss and other systemic symptoms like low-grade fever [3]. Additionally, UC can lead to extraintestinal manifestations (EIMs) affecting the skin, joints, eyes, and liver. The frequency of having at least one extraintestinal joint, ocular, or cutaneous symptom ranges between 6% to 40% [4-6]. Iron deficiency anemia is the most prevalent hematological EIM [7].

Interestingly, a rare but significant correlation exists between UC and immune thrombocytopenia (ITP), a condition characterized by the presence of autoantibodies against platelet membrane antigens leading to immune-mediated platelet destruction. This bleeding condition is defined by isolated thrombocytopenia (platelet count <150,000 u/L) unrelated to systemic illnesses, with an annual incidence estimated at 1 to 6 cases per 100,000 individuals and a prevalence of about 12 per 100,000 cases [8]. The association between ITP and UC, although uncommon, has been documented, suggesting antigenic mimicry where shared or closely related antigens between the gut lumen and the platelet surface could lead to an autoimmune response against platelets in patients with UC [9, 10]. This case focuses on the rare correlation between ITP and UC and highlights the diagnostic difficulties associated with extraintestinal manifestations (EIMs) in individuals with inflammatory bowel disorders (IBD).

## Case presentation

A 26-year-old South Asian male with no significant medical history presented to the GI clinic with a four-week history of hematochezia. He reported initially experiencing one to two episodes of rectal bleeding daily, which later increased to three to four episodes daily, often associated with straining during defecation. The patient also noted having bowel movements approximately one hour after each meal. Notably, he denied other gastrointestinal symptoms such as abdominal pain, melena, nausea, vomiting, diarrhea, pain during defecation, dysphagia, weight changes, or a family history of GI malignancy. He had no remarkable past medical, surgical, or family history, and his social history indicated social alcohol use but no smoking or illicit drug use.

The patient reported that he had previously attributed his rectal bleeding to possible hemorrhoids but had started experiencing abdominal cramping and frank blood without stool. He mentioned his recent travel history, which was domestic with a trip to California. The patient was instructed to go to the emergency department (ED) due to thrombocytopenia noted on recent blood work, which raised concerns about potential brisk bleeding. He presented to the ED with stable vital signs: blood pressure 138/83 mmHg, heart rate 78 beats per minute, respiratory rate 20 breaths per minute, temperature 98.0°F, and oxygen saturation of 98% on room air. On physical exam, bowel sounds were present, the abdomen was soft, non-tender, and without peritoneal signs. No visceromegaly was palpated. No significant rashes or petechia were noted. Digital rectal examination (DRE) revealed no blood or melena, and no external hemorrhoids were observed.

Initial labs on admission showed a white blood cell count (WBC) of 7.08 K/mm^3^, red blood cell count (RBC) of 4.55 M/mm^3^, hemoglobin (HGB) of 12.7 g/dL, hematocrit (HCT) of 39.5%, mean corpuscular volume (MCV) of 86.8 fL, manual platelet count of 18 K/mm^3^, international normalized ratio (INR) of 1.0, prothrombin time (PT) of 12.9 seconds, and partial thromboplastin time (PTT) of 26.9 seconds, erythrocyte sedimentation rate (ESR) of 36 mm/hr (Table 1). Tests for antinuclear antibodies (ANA), rheumatoid factor, and HIV were negative. Peripheral smear revealed normal-looking red blood cells, one giant platelet, normal-looking neutrophils, few atypical lymphocytes, no blasts seen, and no schistocytes or parasites noted. Fecal occult blood testing (FIOBT) was positive. CT scan of the abdomen and pelvis showed mild thickening of the rectosigmoid colon, suggesting colitis but no active gastrointestinal hemorrhage.

**Table 1:** Laboratory findings during the course of the patient’s hospitalization WBC: White blood cells; RBC: red blood cells; HGB: hemoglobin; HCT- hematocrit; MCV: mean corpuscular volume; INR: international normalized ratio; PT: prothrombin time; PTT: partial thromboplastin time; ESR: erythrocyte sedimentation rate

CBC	Admission	Day 2	Day 4	Four days after discharge
WBC (4.50-11 K/mm^3^)	7.08 K/mm^3^	7.42 K/mm^3^	6.97 K/mm^3^	5.66 K/mm^3^
RBC (4.60-6.20 M/mm^3^)	4.55 M/mm^3^	4.51 M/mm^3^	3.83 M/mm^3^	4.33 M/mm^3^
HGB (13.5-18.0 g/dL)	12.7 g/dL	12.5 g/dL	10.6 g/dL	12.0 g/dL
HCT (40.0-54.0%)	39.5%	39.6 %	32.7 %	38.6 %
MCV (80.0-96.0 fL)	86.8 fL	87.8 fL	85.4 fL	89.1 fL
Platelets (150-450 K/mm^3^)	18 K/mm^3^	21 K/mm^3^	85 K/mm^3^	227 K/mm^3^
INR (0.9-1.1)	1.0	NA/-	NA/-	NA/-
PT (9.4-12.5 sec.)	12.9 seconds	NA/-	NA/-	NA/-
PTT (25-37 sec.)	26.9 seconds	NA/-	NA/-	NA/-
ESR (0-15 mm/hr)	36 mm/Hr	NA/-	NA/-	NA/-

The patient was admitted to the medicine service, where he received gentle intravenous fluid hydration and IV proton pump inhibitors twice daily. He also received ciprofloxacin 400 mg IV twice daily and metronidazole 500 mg IV three times daily as empiric antibiotics for colitis. The hematology team recommended starting intravenous immunoglobulin (IVIG) at a dose of 80 grams for two days. Steroids were withheld, considering colitis findings on the CT abdomen.

Throughout his hospital stay, the patient’s platelet count improved from 21 K/mm^3^ to 85 K/mm^3^ after two doses of IVIG, and his vital signs and clinical condition remained stable. Therefore, it was decided to discharge him on oral antibiotics and with outpatient follow-up appointments in hematology and gastroenterology clinics for further evaluation and to schedule a colonoscopy.

**Figure 1:**
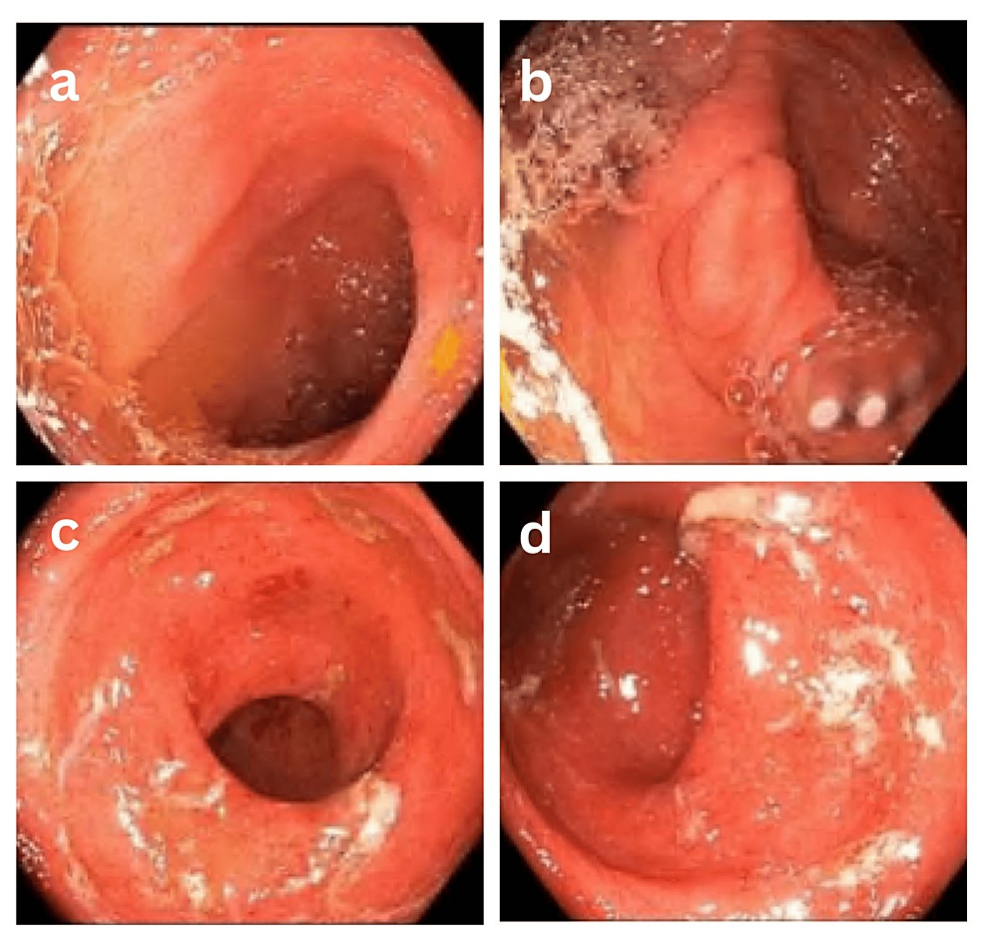
(a) Normal terminal ileum; (b) appendiceal orifice; (c and d) rectum with boggy, erythematous, friable mucosa noted extending from approximately 18 cm from the anal verge. Small internal hemorrhoids were noted at the anal verge.

Four days after discharge, the patient was seen in the GI clinic and reported feeling better than during admission. Follow-up laboratory results showed an improvement in the platelet count to 227 K/mm^3^ and elevated fecal calprotectin at 611 μg/g (normal range <50 μg/g). Subsequently, a colonoscopy was performed, which revealed erythematous, friable mucosa (Figure 1).

Multiple biopsies were obtained via cold biopsy forceps and sent for histopathologic analysis. The report showed mild chronic inflammation in the cecum and ascending colon. Mild chronic inflammation was observed in the sigmoid colon, along with superficial and focal lamina propria congestion and hemorrhage. A biopsy of the rectum demonstrated diffuse active chronic colitis with focal cryptitis, crypt abscesses, and crypt loss, findings which are likely indicative of IBD, specifically UC. Therefore, UC was diagnosed, and ITP was attributed as an extraintestinal manifestation of UC. Treatment was initiated with mesalamine 1600 mg orally three times daily and mesalamine suppositories 1000 mg every night at bedtime. Currently, the patient is actively following up in the gastroenterology clinic, and he reports improvement in his symptoms and good compliance with the medication regimen.

## Discussion

ITP is characterized by an immune-mediated decrease in circulating platelets, manifesting as easy bruising, petechiae, or significant bleeding in severe cases. Its pathogenesis is believed to be driven by autoantibodies targeting platelet membrane glycoproteins, leading to their opsonization and subsequent phagocytosis in the spleen [11]. On the other hand, UC is an inflammatory bowel disease typified by chronic colon and rectum inflammation. The exact etiology remains a topic for research, but it is believed that a combination of genetic susceptibility, environmental factors, and dysregulated immune responses play a pivotal role [3]. 

The appearance of ITP as an extraintestinal manifestation of UC is a rare clinical manifestation. The frequency of ITP among ulcerative colitis patients is estimated to be 0.1-0.48% [12]. It has been posited that the antigenic mimicry between luminal antigens in the gut and platelet surface antigens may serve as a potential mechanism that connects the two conditions. Essentially, the immune system, in trying to combat the luminal antigens associated with UC, may mistakenly target platelets, leading to thrombocytopenia [10]. 

Furthermore, the importance of a thorough evaluation in patients with UC is highlighted as our patient initially presented primarily with gastrointestinal symptoms rather than the overt signs or symptoms typically associated with a significant reduction in platelet count (such as petechiae or significant bruising). It also emphasizes the necessity to not solely attribute gastrointestinal symptoms to common diseases like hemorrhoids, especially when the symptomatology escalates, as observed in our patient.

Moreover, the positive outcome following treatment with intravenous immunoglobulin (IVIG) is consistent with literature suggesting its efficacy in ITP management by saturating the Fc receptors on macrophages, thus reducing platelet phagocytosis [13]. The subsequent diagnosis of UC, based on colonoscopy findings and histopathological evidence, provided the final piece of the diagnostic puzzle. The marked improvement in platelet count and gastrointestinal symptoms following UC-targeted treatments further solidifies the hypothesis of ITP being an extraintestinal manifestation in this case.

## Conclusions

In conclusion, ulcerative colitis (UC) is a recognized inflammatory condition of the colon characterized by specific gastrointestinal symptoms and extraintestinal manifestations affecting various organ systems. These manifestations can significantly impact disease management and patient quality of life. Immune thrombocytopenia (ITP), although rare, has been documented in association with UC, likely due to antigenic mimicry leading to an autoimmune response against platelets. This association highlights the importance of thorough clinical evaluation and ongoing monitoring in UC patients to address both gastrointestinal and extraintestinal manifestations effectively.
